# Increased Heat Absorption by the Walls of Exchangers Sprayed with Coatings Exhibiting High Heat Absorption and Conductivity

**DOI:** 10.3390/ma18194619

**Published:** 2025-10-06

**Authors:** Sławomir Morel, Monika Górska

**Affiliations:** Faculty of Production Engineering and Materials Technology, Czestochowa University of Technology, 42-201 Czestochowa, Poland; slawomir.morel@pcz.pl

**Keywords:** plasma, ceramic coatings, cermet coatings, emissivity of coatings, plasma

## Abstract

The article presents a method for selecting spray coating systems for furnace walls and heat exchangers, aimed at protecting them and intensifying heat exchange processes. Calculations were made of the effect of the mutual emissivity coefficient between the heating medium (exhaust gases) and the surface of the exchanger—both uncoated and coated—on the heat flux value. Selected coating systems were applied in laboratory conditions by spraying them onto the boiler surfaces and then measuring their heat exchange efficiency with the cooling medium (water) flowing through the piping system. The results of the laboratory tests were verified under industrial conditions in metallurgical installations, confirming the accuracy of the calculations and the validity of using spray coatings to increase thermal efficiency. The use of appropriately selected coating systems increases heat absorption, extends the service life of exchangers, reduces the risk of cooling system failure, and lowers the cost of heating equipment repairs.

## 1. Introduction

Heating devices, such as furnaces, boilers and heat exchangers, operate under conditions of high temperatures and intense heat exchange. Their efficiency depends on the properties of the materials from which they are made, as well as the protective technologies used [1,2,3]. The selection of suitable materials and technologies for their manufacture is crucial, as they must meet the requirements for thermal conductivity, strength, and resistance to extreme operating conditions [4,5,6]. One of the most important factors influencing the thermal performance of furnaces is the ability of the surface to absorb and emit heat, which directly translates into the heating efficiency of the charge [7]. In this case, plasma-sprayed coatings are an effective solution to increase the thermal performance of furnaces by optimizing heat transfer [8,9]. They also play a key role in protecting heating equipment by improving its resistance to wear, erosion, and operation in corrosive environments [10,11,12,13].

Radiative heat transfer, which depends on the emissivity and thermal conductivity coefficients of the surface, is an important factor affecting the efficiency of heating equipment. These properties can be modified by applying coatings with specific roughness and porosity of structure [14,15,16,17]. Plasma-sprayed coatings can act as heat-absorbing and heat-emitting layers, allowing more efficient use of thermal energy [18]. By using appropriate materials, these coatings can intensify the absorption of thermal energy from the flame and flue gases, and then efficiently transfer it to the charge inside the furnace [19,20]. Depending on their intended thermal functions, coatings used in heating equipment can be divided into two basic groups:

Heat-absorbing coatings (Group A)—absorb heat radiation and conduct heat toward the substrate.

Heat-accumulating (insulating) coatings (Group B)—radiate stored heat from the surface of the coating.

Absorbent coatings should have high open porosity, which provides a developed surface with high heat absorption capacity [21]. In addition, a high thermal conductivity coefficient (λ) is an important parameter. These coatings are used on the radiant wall surfaces of recuperators and boilers to increase the ability to absorb heat from the flame, gas or flue gases and efficiently transfer it to heated media such as air, water, or steam [9]. This makes it possible to increase the thermal efficiency of equipment and improve its energy performance [22]. In contrast, heat-accumulating coatings should absorb heat at the surface and efficiently radiate it toward the heated load. The key condition for their effectiveness in this case is to limit the penetration of the accumulated heat into the coating—it should only increase the surface temperature [23,24]. Therefore, these coatings must be characterized by high thermal insulation properties and adequate thickness [25,26,27]. They are applied to the walls and vaults of furnaces, especially those operating intermittently, as well as to the walls of flue pipes behind heating furnaces, often with recuperators built into the pipes. Due to the operating environment in which the equipment in question operates, including high temperatures, aggressive flue gases and intense mechanical stresses, these factors can shorten the life of the equipment, causing it to crack, corrode or lose its heat-conducting properties [28,29]. Plasma-sprayed coatings (atmospheric or vacuum) provide an effective solution to this problem, as they allow surface properties to be tailored to specific operating conditions. With the ability to apply layers of the required and desired microstructure [21,30], these coatings are better able to withstand mechanical and thermal stresses than traditional coatings with a continuous—lamellar microstructure [30]. Thus, Group A and B coatings intensify the absorption of heat from the flame and from the flue gas stream leaving the working spaces of furnaces and heat exchangers. Consequently, they reduce the temperature of the flue gases discharged into the chimney, resulting in effective heat recovery and improved thermal efficiency of the equipment. The choice of coating material depends on the function to be performed by the coating and the operating conditions of the equipment [12]. The most commonly used are ceramic oxides (Al_2_O_3_, ZrO_2_, and Cr_2_O_3_), carbides (WC-Co and Cr_3_C_2_-NiCr) and metal alloys (NiCr and NiAl) [25,26,27,28,29,30]. In addition, it has been observed that yttria-stabilized zirconium oxide (YSZ) is particularly valuable in thermal barrier coatings (TBCs) that protect nickel and cobalt alloys from extreme temperatures [31].

In the pursuit of desired coating properties such as porosity, corrosion resistance and thermal conductivity, spray process parameters, including gas flow, input power, distance from the substrate and powder characteristics, play an important role [32]. Variation in these parameters leads to the formation of corresponding structures, partially molten zones and areas of different hardness and elastic modulus. Plasma, which is the fourth state of matter, consists of neutral atoms, ionized ions, and free electrons. The plasma spraying process uses an electric arc to create a plasma jet with temperatures as high as more than 10,000 K, allowing it to melt virtually any powder material [33]. Plasma torches (plasmotrons) focus arc energy in a nozzle, accelerating powder particles and directing them to the surface of the substrate. There they form a coating layer through rapid cooling (up to 10^6^ K/s), resulting in fine structures 20 to 200 µm thick [34]. With the Atmospheric Plasma Spraying (APS) process, coatings of 0.1 to 1.0 mm thickness can be applied either manually or with industrial robots, ensuring high repeatability and precision. This makes it possible to coat surfaces with complex geometries and control the angle of impact of particles on any surface.

The use of plasma-sprayed coatings in heating systems has long been of interest to researchers around the world [35]. In the face of growing energy and environmental challenges, plasma spray technology appears to be one of the most promising solutions to support the transformation of the thermal industry towards efficiency and sustainability. This technology enables precise shaping of thermophysical properties of surfaces, such as emissivity, thermal conductivity, and corrosion resistance, which translates into improved thermal efficiency of equipment and its operational life [18]. As noted by numerous authors, the reduction in thermal losses through the use of coatings with optimized microstructure and precisely selected material composition leads to a significant reduction in energy demand, which directly translates into lower operating costs and reduced greenhouse gas emissions [22]. In the countries of the European Union, economic pressures resulting from high wholesale energy prices and the cost of purchasing CO_2_ emission allowances are becoming a factor stimulating the implementation of energy-saving technologies. Consequently, there is a need to seek solutions to reduce their impact on the wider environment. Plasma-sprayed coatings, thanks to their application versatility, are used in a variety of configurations of heating equipment from industrial furnaces to heat exchangers while acting as absorbing, emitting, or insulating layers. Their presence on working surfaces makes it possible not only to increase energy efficiency, but also to significantly reduce the degradation of base materials due to thermal, mechanical, and chemical factors. In the context of heat exchangers, ceramic and carbide coatings are currently the most commonly used, mainly due to their availability, relatively low price, and resistance to corrosion and high operating temperatures. They are characterized by high heat absorption capacity due to their rough surface, high hardness and mechanical durability, limited thermal conductivity and decreasing emissivity with the addition of a metal component.

The main objective of the work was to determine the extent to which the method of applying spray coatings and the selection of specific metallic and ceramic coatings improve the thermal efficiency and performance of heating devices. In particular, the aim was to develop coating systems for application to the surfaces of furnace walls and heat exchangers, whose protective properties promote intensified heat exchange and extend the service life of structural elements. Despite promising results, metal alloy coatings are much less frequently studied than their ceramic counterparts: Ti_2_C_3_, WC + Co, TiN, AlMoFe, MnO_2_, and CeO_2_. Laboratory experiments to date indicate their great potential, but the scientific literature remains limited. New research can add to existing knowledge and contribute to the development of more efficient heat transfer solutions in industrial implementations. The main objective of the study was to determine to what extent the method of spraying and the selection of suitable material for the coating of a given type of thermal equipment improves its operation and thermal efficiency.

## 2. The Issue of Selecting Materials for Coatings

The first factor to consider when undertaking research in this area is the absorption capacity of metals and ceramics used for spraying coatings onto selected heating device components.

Furnaces, boilers, recuperators, and other large-size heating devices operate in elevated and high temperatures, where radiant heat exchange is the key mechanism of energy exchange. Its efficiency depends largely on the properties of working surfaces, in particular on the values of emissivity and thermal conductivity coefficients. These parameters can be modified by applying appropriately designed coating systems to the irradiated surfaces of heating equipment walls [36,37,38].

**Figure 1:**
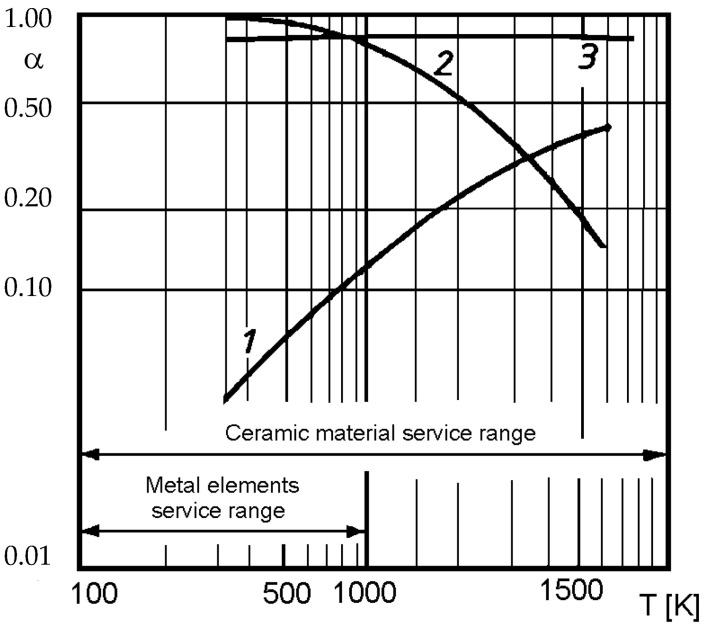
Variation in surface absorptivity and emissivity depending on temperature of bodies for different materials. Reprinted from Ref. [40,41]. (1—metal; 2—non-metal; 3—coating (coarse—ceramic or cermet)).

Coating systems are shaped by the selection of material composition (ceramics, metallic additives), component grain size, and plasma spraying process parameters. Key characteristics such as thickness, surface roughness, and structure porosity determine their thermal and mechanical properties. When selecting materials for spraying, it is important to analyze the absorption capacity of metals and ceramics, which determine the thermal efficiency of coatings and their suitability for specific industrial applications. In industry, many methods are used to give heating agents (exhaust gases, flames) high emissivity, such as flame carburization [39,40]. Less well known and less frequently used are methods of giving irradiated walls and ceilings of furnaces, flue gas ducts, and walls of heat exchangers a high capacity for absorbing and emitting thermal radiation. The heat exchange flux depends on the mutual emissivity coefficient, which is a function of the emissivity coefficients of the heating and heated walls. The values of the heat absorption and emission coefficients of materials commonly used in the construction of furnaces and thermal devices (metal or ceramic) and coatings are shown in Figure 1 [39,40].

Based on the data presented in Figure 1, it can be seen that the absorptivity of metals increases approximately linearly with increasing temperature, while ceramic materials show the opposite trend. It also shows that the emissivity coefficient of ceramic and cermetal (rough) coatings has a high and constant value. However, it is important to note that parameters such as the roughness of metal surfaces, contamination, and corrosion products increase the emissivity coefficient so that its value is analogous to that of ceramics, i.e., higher than 0.7. Due to the complexity of heat transfer through coatings and the dependence of these processes on a large number of parameters, there is a lack of equations in the literature that would allow the calculation of ε i λ depending on the type of coating and spraying parameters [39,40]. In view of the above, there was a need to develop methods for testing the emissivity and thermal conductivity of multilayer coatings [41,42], testing thermal parameters, and selecting coatings for spraying furnace walls, heat exchangers, and other heating devices while ensuring the efficiency and quality of these devices.

## 3. An Analytical Approach to Determining the Optimal Value of the Mutual Emissivity Coefficient (ε_ₚ_) Depending on the Amount of Absorbed Heat

The starting point for considerations regarding the selection of the mutual emissivity coefficient (εₚ) in the context of the amount of heat absorbed, in accordance with the subject matter under investigation, is the relationship described by Formula (1), which defines the amount of thermal energy exchanged between the heating medium and the surface of heat exchanger walls or other thermal devices covered with a sprayed coating.

(1) $$Q_{prom}+Q_{konw}=Q_{prz}$$

(2) $$F_{s}\varepsilon_{w}\sigma_{c}\left(T_{g}^{4}-T_{s}^{4}\right)+F_{s}\alpha_{k}\left(T_{g}-T_{s}\right)=F_{s}\frac{\left(T_{s}-T_{n}\right)}{R_{p}}$$

Equation (2) defines the mutual emissivity coefficient εₚ in accordance with the relationship presented in Equation (2a), the correctness of which is based on the assumption that exhaust gases—acting as a heating medium—can be treated as a gray body.

(2a) $$\varepsilon_{w}=\frac{1}{\frac{1}{\varepsilon_{g}}+\frac{1}{\varepsilon_{s}}-1}$$

The thermal resistance value of the exchanger wall with a sprayed coating system is determined by Equation (2b)

(2b) $$R_{p}=\frac{x_{w}}{\lambda_{w}}+\frac{x_{pod}}{\lambda_{pod}}+\frac{x_{s}}{\lambda_{s}}+\frac{1}{\alpha_{n}}$$

By substituting (2a) and (2b) into Equation (2), Equation (3) is obtained.

(3) $$F_{s}\frac{1}{\frac{1}{\varepsilon_{g}}+\frac{1}{\varepsilon_{s}}-1}\sigma_{c}\left(T_{g}^{4}-T_{s}^{4}\right)+F_{s}\alpha_{k}\left(T_{g}-T_{s}\right)=F_{s}\frac{\left(T_{s}-T_{n}\right)}{\frac{x_{w}}{\lambda_{w}}+\frac{x_{pod}}{\lambda_{pod}}+\frac{x_{s}}{\lambda_{s}}+\frac{1}{\alpha_{n}}}$$

Formula (3) shows that the following factors are decisive for the rational use of heat from heating agents (flames or exhaust gases):

− high temperature of heating agents,
− high emissivity of heating agents,
− high emissivity of walls (furnaces and devices) participating in radiant heat exchange,
− high thermal conductivity or insulation of walls participating in heat exchange.

In order to assess the impact of the wall emissivity coefficient εs on the mutual emissivity coefficient εw, calculations were performed according to Equation (2a), assuming a constant emissivity value of the heating medium (exhaust gases) **ε_g_** = 0.40. The value of the wall emission coefficient **ε_s_** was assumed to be in the range from 0.20 to 0.50 for metal walls, through 0.55–0.65 for ceramic walls (refractory materials) and from 0.70 to 0.95 for coating systems made of metal oxides, carbides, and other mixtures used in the tests. 

**Figure 2:**
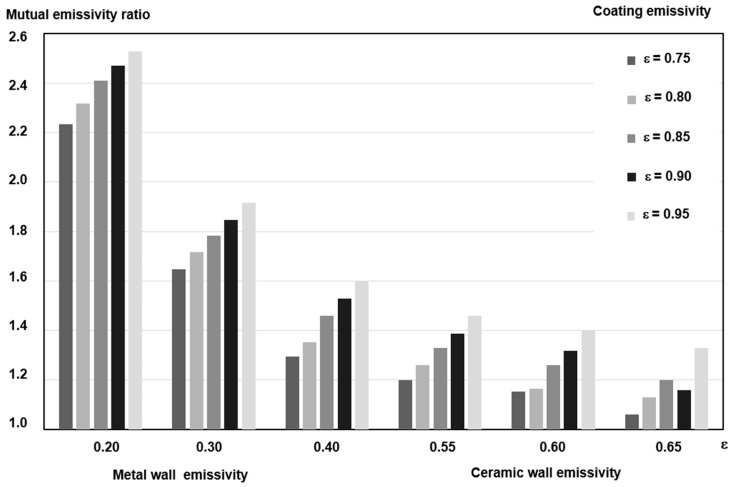
Distribution of mutual emissivity coefficients: metal wall without coating system and with coating system and ceramic wall without coating system and with coating system.

Based on the adopted emission coefficient values for metal, ceramic, and coating structures, extended heat exchange calculations were performed, the results of which are presented in Figure 2.

The mutual emissivity coefficient was calculated for the heating medium—uncoated metal wall system (ε_w.b.pow_), and then the mutual emissivity of the heating medium—coated wall system (ε_w.z.pow_) was calculated, assuming values of ε_pow_ = 0.90 for the coating system, after which the value of the mutual emissivity coefficient was determined—heating medium—wall with coating system to mutual emissivity, heating medium—wall without coating.

$$\frac{\varepsilon_{w.z.pow}}{\varepsilon_{w.b.pow}}$$

For the metal wall of the boiler or the pipes of the recuperator segment (generally—diaphragm heat exchanger), ε_s.met_ = 0.30 and ε_g_ = 0.40 were assumed. The value of the mutual emissivity coefficient of the wall without coating (ε_w.b.pow_), calculated from Formula (2a), is:

(4) $$\varepsilon_{w}=\frac{1}{\frac{1}{0.40}+\frac{1}{0.30}-1}=0.207$$

For the same metal surface of the exchanger, but with a sprayed coating system with an emissivity coefficient of ε_pow_ = 0.90, the mutual emissivity value (calculated according to Formula (2a)) is:

(5) $$\varepsilon_{w}=\frac{1}{\frac{1}{0.40}+\frac{1}{0.90}-1}=0.383$$

The ratio of the mutual emissivity coefficient of the flame and the wall with a sprayed coating to the mutual emissivity of the flame and the wall without a coating is:

(6) $$\frac{\varepsilon_{w,zpow}}{e_{w,bpow}}≅1.851$$

The result obtained represents an 85% increase in the amount of heat absorbed by the metal surface of the exchanger with a sprayed coating compared to the surface of the exchanger without a coating. The mutual emissivity coefficient described by Formula (2a) has a decisive influence on the amount of heat exchanged by radiation. This effect is more intense the lower the absorption capacity of the irradiated wall (before spraying with the coating system). Therefore, in the operation of devices with metal walls, the presence of a coating system with a high emissivity value ε_s_ significantly increases the heat exchange flux. The results of measurements of the thermal and mechanical properties of cermet coatings based on oxides: aluminum, chromium, and zirconium with the addition of NiAl, as well as coatings made of a mixture of Cr_3_C_2_ + NiAl, are presented in Table 1**.**

The study examined three mechanical properties of coatings (thermal shock resistance, erosion resistance, and gas permeability) and two thermal parameters: emissivity and thermal conductivity.

The methodology used to measure these parameters was developed at the Plasma Thermology Department of the Czestochowa University of Technology and is described in other articles by the authors. These are qualitative methods, e.g., resistance to thermal shocks determined on the basis of the number of heating cycles in a furnace of a sample with a coating to a temperature of 700 K and cooling it in air. After cooling in air, the condition of the coating was checked; if the coating did not crack, it was put back into the furnace and heated to 700 K. After reaching this temperature, the sample was removed and cooled. These steps were repeated until the coating cracked—the number of heating-cooling cycles was a measure of thermal shock resistance.

Erosion resistance was determined by measuring the time it took for the coating to lose 1 g of its mass during sandblasting (abrasive blasting) of a sample with a sprayed coating.

The gas permeability of the coating was determined by measuring the pressure to which gas (air) could be compressed in a tank separated by a ring with a layer of coating detached from the tank base filled with water, allowing the flow of gas through the coating layer to be determined.

The method described in Patent No. 163658 was used to determine the emission coefficient, and the apparatus described in Patent No. 164710 was used to determine the thermal conductivity of the coatings.

The results of the elements implemented and tested in industry were determined on the basis of heat balances and observations of their operating time.

The described methodology for evaluating coatings was not covered in this article and has been described in other articles by the authors [40,41]. and has been patented and is reserved and protected by the Plasma Thermics Department of the Częstochowa University of Technology. Table 1 summarizes the mechanical and protective properties (columns 3–5), thermal properties (columns 6–7), and column 9 provides a point-based assessment of the suitability of cermetal coating systems for spraying heat exchanger walls.

Columns 3 to 5 present the key protective properties of the tested coatings, which were evaluated using a weighted scoring method. Each property was classified on the OPS scale (from 0 to 12), i.e., Statistical Coating Assessment, which refers to a point-based system for evaluating the properties of protective coatings used in technical and material analyses, where OPS = 0 indicates the lowest quality of properties and OPS = 12 indicates the highest quality of properties. Column 7 shows the heat radiation absorption capacity in the form of a dimensionless OPS value, i.e., 0.7 was subtracted from the actual emission coefficient value and multiplied by 100, calculated according to the formula:

OPS _absorption_ = (ε_actual_ − 0.7) × 100

For example, for an emission coefficient ε = 0.92.

OPS _absorption_ = (0.92 − 0.7) × 100 = 22

This value reflects the absorption efficiency of the coating in terms of heat e change. The total value is given in column 9, which is the sum of the points from columns 6 + 7 + 8. This value is a synthetic indicator of the quality of the coating for use in diaphragm heat exchangers, indicating the optimal combination of functional properties.

Based on the analyses conducted, it was found that cermet coating systems based on chromium carbide (Cr_3_C_2_) demonstrated the highest efficiency in heat exchanger surface spraying applications, scoring approximately 70 points on the agreed assessment scale. The second place was taken by coatings made of mixtures of chromium oxide (Cr_2_O_3_) and nickel aluminide (NiAl), achieving a score exceeding 50 points. The third place was taken by coating systems based on mixtures of aluminum oxide (Al_2_O_3_) and nickel aluminide. On the other hand, coatings made of mixtures of zirconium oxide (ZrO_2_) and NiAl proved to be the least useful in the context of applications on the surfaces of boilers, recuperators, and other thermal devices. The results of the calculations served as a criterion for the selection of appropriate coatings sprayed onto the surfaces of thermal devices, due to their impact on the intensity of heat exchange. The validity of the analytical tests was confirmed in an experiment. The selected coatings were applied to specific elements of heat exchangers, and then measurements were taken of their effectiveness in terms of heat flux values.

## 4. Research Position

**Figure 3:**
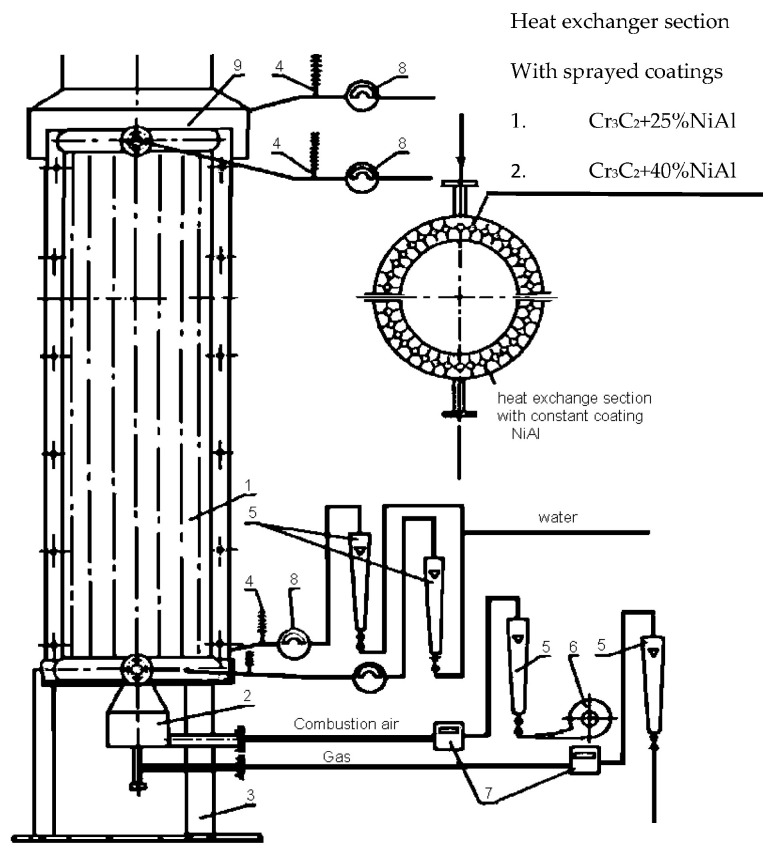
Schematic diagram of installation of model heat exchanger used for testing effect of coating types on heat exchange in waste heat boiler. Reprinted from Ref. [39,40] (1—model waste heat boiler; 2—burner; 3—stand; 4—thermocouple–recorder; 5—rotameter; 6—fan; 7—gas meter; 8—water meter; 9—hood with combustion gas exhaust and thermocouple for measurement of combustion-gas temperature).

The heating chamber used in the tests allowed for the simulation of conditions similar to those prevailing in industrial heating devices on a laboratory scale. A diagram of the test stand on which the measurements of the impact of coating systems were made is shown in Figure 3. The test stand (boiler model) is located at the Czestochowa University of Technology at the Faculty of Mechanical Engineering and is an original design by S. and Sł. Morel.

**Figure 4:**
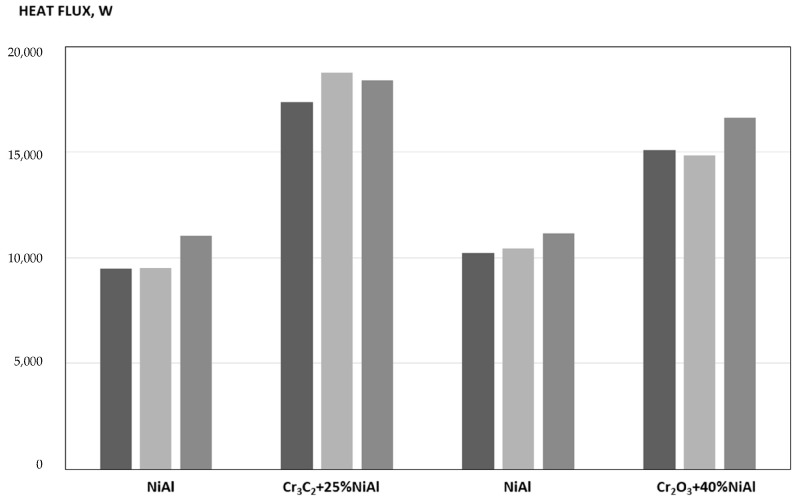
Effectiveness of effect of cermet coatings on flux of heat absorbed by boiler (results of three tests for each variant).

The boiler model shown in Figure 3 was made of two half-cylinders with a radius of 600 mm and a height of 2300 mm. The stand is constructed in a pipe-fin-pipe arrangement (pipes − ϕ 38 × 4, fins − 20 × 4) forming a closed cylinder with a diameter of 1200 mm. Inside the cylinder, natural gas was burned with air using a diffusion burner, producing a flame that reached 3/4 of the boiler’s height. Measurements of heat exchange parameters began after thermal equilibrium was achieved. The tests were carried out in two stages, each consisting of three measurement series. The measurement results were presented graphically in Figure 4 and summarized in Table 2. In the first stage, two half-cylinders with sprayed coating systems were used: half-cylinder 1 sprayed with NiAl (base measurement condition) and half-cylinder 2 sprayed with Cr_3_C_2_ + 25% NiAl. In the second stage, a 1-NiAl half-roller and a 3 half-roller were used, which were sprayed with a coating system consisting of Cr_2_O_3_ + 40% NiAl.

## 5. Analysis of the Results Obtained from Experimental Research

The results of experimental studies determining the impact of selected coatings (NiAl, Cr_3_C_2_ + 25% NiAl, Cr_2_O_3_ + 40% NiAl) sprayed onto irradiated boiler walls in the context of heat transfer efficiency by water in the boiler piping system are presented in (Table 2).

In the first stage of measurements, the most favorable results were obtained in the second series of measurements. The temperature of the water flowing into both half-rollers, i.e., half-roller 1 covered with a coating system (one layer of coating) with NiAl and half-roller 2 covered with a coating system with Cr_3_C_2_ + 25% NiAl, was 11.8 °C, while the temperature at the outlet of the section system was: from half-roll 1—26.0 °C, and from half-roll 2—32.6 °C. 

The increase in water temperature reached: 14.2 °C in half-cylinder 1 and 20.8 °C in Section 2. With the water flow values as given in Table 2, column 8, row 2, the heat flows carried away with the heated water reached the following values:

− from half-cylinder 1 sprayed with NiAl coating—9496 W (100%),
− from half-cylinder 2 sprayed with Cr_3_C_2_ + 25%NiA1 coating—18,779 W (198%), which is twice the value obtained in half-cylinder 1 sprayed with NiAl.

Doubling the amount of heat transferred by water achieved by spraying half-cylinder 2 with a chromium carbide coating system (high **ε** value and high λ_pow_ value of this coating system). The value obtained confirms the high suitability of this type of system for intensifying heat transfer processes from heating agents (exhaust gases) and demonstrates the high efficiency of heat utilization in the working space of an exchanger sprayed with an absorption-conductive coating system.

The increase in heat transfer by water flowing in a pipe system coated with a Cr_3_C_2_ + 25% NiAl coating system in the range from 66.7% to 97.7% reduces the temperature of the exhaust gases, which leads to a reduction in the volume of exhaust gases, and in the case of industrial installations, it increases the concentration of dust in the exhaust gases, which results in an increase in the amount of dust captured (precipitated) in the dust collector through which the exhaust gases flow.

In the second stage of the research, which was carried out by combining half-cylinder 1 (NiAl) with half-cylinder 3 (Cr_2_O_3_ + 40% NiAl), the experimental results showed that the highest values of heat flux carried away with water were achieved in the 6th measurement series (II.6). The heat flux values obtained for water from the exchanger section coated with Cr_2_O_3_ + 40% NiAl were approximately 50% higher than the heat flux values for water flowing out of half-cylinder 1.

The use of cermet coatings made from oxide mixtures containing NiAl proved to be significantly less advantageous than the use of coatings made from carbide mixtures with NiAl.

The developed solution has been implemented in industry. An example of a properly selected research direction are the results confirmed in the industrial operation of furnace vault support beams and batch window frames, summarized in Table 3.

## 6. Conclusions

1. The selection of appropriate spray coatings to protect furnace components and heat exchangers is a key factor in extending the service life of heating equipment, improving its energy efficiency, and reducing its negative impact on the environment. In the face of rising energy costs, rational thermal energy management is becoming increasingly important. Instead of focusing on acquiring new energy sources, it is necessary to conduct intensive research and development work aimed at modernizing existing equipment and optimizing its operating parameters. Furthermore, it has been proven that the systematic implementation of coating spraying technology in equipment such as heat exchangers, waste incinerators, and metallurgical industry installations—one of the most energy-intensive industries—can lead to better energy utilization, reduced heat loss, and reduced pollutant emissions.
2. Experimental studies have confirmed that both ceramic and cermet coating systems with a rough surface have a high heat absorption capacity. The emissivity of coating systems decreases with the addition of a metal component to the ceramic. At the same time, it has been established that the emissivity of metal coating systems exceeds the emissivity coefficient of the same metals in the solid state by more than two times.
3. The use of Cr_3_C_2_ + 25% NiAl spray coatings instead of the base NiAl system significantly increases the thermal efficiency of heat exchangers. The tests carried out showed that thanks to the use of an absorption-conductive post-coating system, it was possible to double the amount of heat transferred to the working medium (water) compared to the base state.
4. The increase in heat transfer efficiency from 66.7% to 97.7% confirms the high effectiveness of chromium carbide coatings in intensifying heat exchange with exhaust gases. This results in a reduction in exhaust gas temperature, which leads to a decrease in their volume and, in industrial conditions, to an increase in the concentration of dust in the waste gases. This phenomenon contributes to increased dust removal efficiency by intensifying the process of solid particle precipitation in dust collectors.
5. Studies conducted on the impact of spray-applied coating systems on equipment components used in laboratories and industrial conditions have shown that in the presence of cermet coating systems with high heat emission and conduction values, there was an increase in the amount of heat carried away by the heated media flowing in the exchangers (water or air).
6. Furthermore, it has been established that the values of the emissivity coefficient of the outer layer of the coating system correlate with the relationship e_w_ = 1/(1/e_g_ + 1/e_s_ − 1) and affect the value of the heat flux absorbed by the heated working fluids flowing in the boiler with coatings formed on their surfaces.

## Figures and Tables

**Table 1:** Point-based assessment of the suitability of coating systems for heat exchangers, furnaces, and other thermal equipment.

No.	Coating Type	Protective Properties				Thermal Properties		Suitability for Spraying
		Thermal Shock Resistance	Erosion Resistance	Internal Porosity	Total Points	Heat Radiation Absorption Ability {ε}	Thermal Conductivity {λ}	Membrane Heat Exchangers
							W m^−1^ K^−1^	
1	2	3	4	5	6	7	8	9
		Number of temperature change cycles	Points for 1 g coating loss time	Points for gas pressure equalization time	3 + 4 + 5	1 point for each 0.01 above 0.7	actual value of λ	6 + 7 + 8 for *p* > 40 is good
1	Al_2_O_3_	0	10	6.8	17	16 (0.86)	3	36
2	Al_2_O_3_ + 10% NiAl	2	10	6.0	18	13	9	40
3	Al_2_O_3_ + 25% NiAl	6	10	6.5	23	9	13	45
4	Al_2_O_3_ + 40% NiAl	9	5	5.4	19	6	16	41
5	Cr_2_O_3_	0	8	6.3	14	22 (0.92)	5	41
6	Cr_2_O_3_ + 10% NiAl	4	8	6.0	18	16	9	43
7	Cr_2_O_3_ + 25% NiAl	4	7	5.7	17	12	19	48
8	Cr_2_O_3_ + 40% NiAl	8	6	5.0	19	9	21	49
9	ZrO_2_	0	1	11.5	13	15 (0.85)	1	29
10	ZrO_2_+10% NiAl	1	2	10.0	13	10	4	27
11	ZrO_2_ + 25% NiAl	5	3	8.0	16	5	7	28
12	ZrO_2_ + 40% NiAl	7	4	7.2	18	2	9	29
13	Cr_3_C_2_ + 25% NiAl	11	12	4.5	28	13 (0.93)	29	70
14	Cr_3_C_2_ + 40% NiAl	12	11	4.0	27	10	32	69

**Table 2:** Parameters of water flows passing through boiler sections.

Stage Series	Coating Type	Burner Feed Parameters		Flowing Water Parameters				Heat Flux Carried by Water
				Temperature		Temperature	Mass	
		Gas	Air	Inlet	Outlet	Increase	Flux	
		m^3^h^−1^	m^3^h^−1^	°C	°C	°C	g s^−1^	W
**1**	**2**	**3**	**4**	**5**	**6**	**7**	**8**	**9**
**I.1**	NiAl	2	18	11.1	24.9	13.8	164	9460
	Cr_3_C_2_ + 25% NiAl			11.1	30.9	19.8	210	17,380
**I.2**	NiAl	2	18	11.1	26.0	14.2	160	9496
	Cr_3_C_2_ + 25% NiAl			11.1	32.6	20.8	216	18,779
**I.3**	NiAl	2	18	11.1	25.5	14.5	182	11,031
	Cr_3_C_2_ + 25% NiAl			11.1	30.5	19.5	226	18,421
**II.4**	NiAl	2	18	11.1	25.0	13.9	176	10,226
	Cr_2_O_3_ + 40% NiAl			11.1	30.3	19.1	189	15,089
**II.5**	NiAl	2	18	11.1	26.1	14.7	170	10,445
	Cr_2_O_3_ + 40% NiAl			11.1	29.8	184	193	14,844
**II.6**	NiAl	2	18	11.1	25.1	14.1	189	11,140
	Cr_2_O_3_ + 40% NiAl			11.1	30.4	19.4	180	16,637

**Table 3:** Comparison of laboratory and industrial results of coating impact.

No.	Coating Characteristics				Obtained Results							
	Coating Description	Composition	Thermal Parameter Values		Effects Achieved on Laboratory Stands					Effects Achieved in Industrial Conditions		
			ε_T_	λ Wm^−1^K^−1^	Without Coating W		With Coating W		Increase in Thermal Efficiency	Element Name	Obtained Result, incl.	
											Thermal Effects	No. of Campaigns Endured
1	2	3	4	5	6		7		8	9	10	11
1	Absorptive and conductive	Cr_3_C_2_ + 25% NiAl	0.75	30	9490		173,780		84%	furnace roof supporting beams	30% increase in steam amount	3 campaigns
2	Absorptive and conductive	Cr_3_C_2_ + 25% NiAl	0.75	30	9490		17,380		84%	charging door frames	30% increase in steam amount	3 campaigns
3	Absorptive and conductive	Cr_3_C_2_ + 40% NiAl	0.86	20	10,226		15,089		48%	boiler hopper	in operation	
4	Absorptive and conductive	Cr_3_C_2_ + 40% NiAl	0.86	20	10,226		15,089		48%	heat treatment furnace crucible	in operation	
5	Absorptive and insulating	int. ZrO_2_ out. Cr_2_O_3_	- 0.82	1 5	t_air_ t_burn._	89 °C 184 °C		104 °C 172 °C	23% 6%	recuperator segment	in operation	

Spraying the radiated surfaces of individual structural elements of metallurgical furnaces resulted in a several dozen percent increase in the amount of steam and its parameters (pressure), as shown in Table 3, rows 1 and 2.

## Data Availability

The original contributions presented in this study are included in the article. Further inquiries can be directed to the corresponding author.
